# Screening of repeated dose toxicity data in safety evaluation reports of cosmetic ingredients issued by the Scientific Committee on Consumer Safety between 2009 and 2019

**DOI:** 10.1007/s00204-020-02868-2

**Published:** 2020-08-24

**Authors:** Emma Gustafson, Christophe Debruyne, Olga De Troyer, Vera Rogiers, Mathieu Vinken, Tamara Vanhaecke

**Affiliations:** 1 Department of In Vitro Toxicology and Dermato-Cosmetology, Vrije Universiteit Brussel, Brussels, Belgium; 2 WISE Lab, Vrije Universiteit Brussel, Brussels, Belgium

**Keywords:** Safety evaluation, Cosmetics, Alternative methods, Repeated dose toxicity

## Abstract

A focal point in the safety evaluation of cosmetic ingredients includes oral repeated dose toxicity testing, which is intended to address the most complex human endpoints. Seven years after the full implementation of the animal testing ban for cosmetic ingredients in the EU, there are still no alternative methods available capable of fully replacing oral repeated dose toxicity testing. Until this issue is resolved, the development of new cosmetic ingredients remains seriously hampered. The present paper describes a thorough screening of the oral repeated dose toxicity data included in safety evaluation reports of cosmetic ingredients addressed in the Annexes of the Cosmetics Regulation (EC) No 1223/2009, issued by the Scientific Committee on Consumer Safety between 2009 and 2019. The liver and the haematological system were identified as the potentially most frequently affected organs upon oral administration of cosmetic ingredients to animals. Evaluation of altered biochemical, morphological, and histopathological parameters related to hepatotoxicity indicated that the most recurrent events are liver weight changes, elevated liver enzymes, and alterations in serum cholesterol and bilirubin levels. Combined listing of affected parameters associated with steatosis and cholestasis indicated the possible occurrence of cholestasis, provoked by a limited number of cosmetic ingredients. The most frequently affected parameters related to the haematological system were indicative of anaemia. An in-depth analysis allowed characterisation of both regenerative and non-regenerative anaemia, pointing to direct and indirect haematotoxicity, respectively. The results presented in this study call for prioritisation of research targeted towards the development of new approach methodologies fit for animal-free repeated dose toxicity evaluation of cosmetic ingredients.

**Electronic supplementary material:**

The online version of this article (10.1007/s00204-020-02868-2) contains supplementary material, which is available to authorized users.

## Introduction

The procedure for safety evaluation of cosmetic products and their ingredients has undergone major changes in Europe during the past decade. Since 11 September 2004, the 7th Amendment to Directive 76/768/EEC prohibits animal experiments of finished cosmetic products, while such testing ban on cosmetic ingredients, or combinations thereof, entered into force from 11 March 2009 onwards (EU 1976, 2003). At the same time, a marketing ban (i.e., the marketing in the EU of cosmetic products and their ingredients tested on animals to evaluate their safety) was introduced for all human health effects. The most complex endpoints, namely repeated dose toxicity (RDT), reproductive toxicity, and toxicokinetics, were exempt until 11 March 2013. The ban was taken up in Cosmetics Regulation (EC) No 1223/2009 (EU 2009), despite the fact that non-animal methods capable of assessing those particular toxicological endpoints were lacking.

Today, validated non-animal methods are available for testing short-term and local toxicological endpoints, including skin irritation/corrosion (1OECD 439, 431, 435, 430) and eye damage/irritation (OECD 491, 492, 437, 438). Additionally, there are validated in vitro/in chemico testing methods for skin sensitisation, some of which have obtained regulatory acceptance as integrated testing approaches (OECD 442D, 442C, 442E, 256). However, 7 years after the full implementation of the animal testing ban for cosmetic ingredients, non-animal methods for testing RDT [i.e., sub-acute (28 days), sub-chronic (90 days), and chronic (85% of expected lifetime) studies], are still lacking.

According to Cosmetics Regulation (EC) No 1223/2009 (EU 2009), the margin of safety (MoS) should be calculated to assess whether a defined exposure to a certain cosmetic ingredient can be considered safe. Despite the fact that the oral route is not the intended, nor expected, route of exposure for the majority of cosmetic products, it is used as a worst-case scenario in the safety assessment unless robust dermal toxicity data are available (SCCS 2018). The MoS is calculated as the ratio of the point of departure_sys_ (POD_sys_) over systemic exposure dose (SED). POD_sys_ denotes the point of departure for systemic toxicity of the substance under consideration. Typically, the no observed adverse effect level (NOAEL) in the most sensitive organ, historically derived through in vivo RDT testing, is used as POD and consequently is a key point in the overall safety assessment of the cosmetic ingredient. The SED is a function of the concentration present in the cosmetic product, usually topically applied, and its dermal absorption. When the MoS is equal to, or greater than, 100, the substance is generally considered safe under the intended use conditions (SCCS 2018).

Several EU and industry-funded projects, both completed and ongoing, such as the Safety Evaluation Ultimately Replacing Animal Testing (SEURAT),^2^ the Integrated European ‘Flagship’ Programme Driving Mechanism-based Toxicity Testing and Risk Assessment for the twenty-first century (EU ToxRisk)^3^ and Cosmetics Europe’s “Long Range Science Strategy” programme, are devoted to finding non-animal approaches capable of addressing questions that historically have been answered through RDT testing (Gocht et al. 2015; Daneshian et al. 2016; Desprez et al. 2018; Vinken 2020). It has been proposed that the breakdown of questions addressed by in vivo RDT testing into smaller components would greatly facilitate the development of suitable non-animal methods for their replacement (Laroche et al. 2019). In this context, identification of target organs of RDT (i.e., the organ where the critical effect occurs) as well as the potential toxicological pathways involved are crucial factors. A pragmatic approach to collect such information is by screening safety evaluation reports, or so-called opinions, issued by the Scientific Committee on Consumer Safety (SCCS). Such safety evaluations are performed for Annex substances of Cosmetics Regulation (EC) No 1223/2009, which are cosmetic ingredients where some concern exists with respect to human health (e.g., colourants, preservatives, UV filters, and hair dyes) (EU 2008, 2009). In each opinion, the SCCS addresses direct questions regarding the safety of the specific cosmetic ingredient and performs a safety evaluation of the ingredient with reference to the intended use (SCCS 2018). In the current study, data from the safety evaluations published by the SCCS between 2009 and 2019 were collected with the specific aim to identify potential target organs of RDT and manifestations of the toxicity in those organs.

## Materials and methods

The study material consisted of 114 SCCS opinions issued between 22 January 2009 and 31 December 2019 dealing with 101 unique cosmetic ingredients in total. All information used in this study was downloaded from the SCCS website.^4^ Hence, no confidential data were used.

For identification of target organ(s), data were manually collected from oral RDT studies described in the SCCS opinions, thus without access to the raw data submitted to the SCCS. The information was sorted into a Microsoft^®^ Office Excel spreadsheet based on the organ in which the critical effect(s) occurred and from which a NOAEL could be derived. In cases of multiple target organs, the sorting was based on what appeared to be the most relevant and sensitive effect. Further analysis of the data included the listing of all changes in morphological, histopathological, and blood biochemical parameters relevant for the top 2 affected organs and/or organ systems described in 88 opinions containing RDT data.

## Results and discussion

### Identification of repeated dose toxicity studies described in the safety evaluation reports

Among the 101 substances covered in the SCCS opinions included in the study, no adequate oral RDT data for determination of an NOAEL were available for 13 cosmetic ingredients. For 1 of the remaining 89 substances, a read-across approach from other routes of exposure was applied. Hence, 88 opinions contained oral RDT data for which an NOAEL determination and subsequent MoS calculation was feasible (Table 1).

**Table 1:** Identification of repeated dose toxicity studies described in 88 safety evaluation reports published between 2009 and 2019

Repeated dose toxicity (oral)	Number of studies	Number of cosmetic ingredients
28-day toxicity study	37	26
90-day toxicity study	110	79
Teratogenicity study	120	79
Two-generation reproductive toxicity study	16	13
Chronic toxicity study	15	9
Carcinogenicity study	30	16

A total of 37 28-day oral toxicity studies were available for 26 cosmetic ingredients, indicating that a particular chemical compound may have been subjected to a similar test more than once. For 19 of these 26 ingredients, also 90-day oral toxicity studies were available. However, in many of these cases, the 28-day study was used as a dose range-finding study for the subsequent 90-day study. In total, there were 110 90-day studies available for 79 cosmetic ingredients. Some of the evaluated cosmetic ingredients have a long history of use and/or have been used in areas other than the cosmetics field, which might contribute to cases where multiple studies have been conducted for the same ingredient. In opinions lacking 90-day oral RDT studies, supporting information was provided from open literature (2) and through read-across from 28-day studies (5) or from chronic studies (2). A total of 120 teratogenicity studies were provided for 79 cosmetic ingredients, again suggesting that a specific ingredient may have been repeatedly subjected to similar tests. Because teratogenicity studies are specifically designed to assess the effects on maternal health, foetal abnormalities and/or altered growth of the foetus, they were not further considered in the present study. Chronic, carcinogenicity, and two-generation reproductive toxicity studies were represented to a lesser extent in the SCCS opinions, albeit displaying a similar pattern of studies repeated for a certain ingredient.

### Description of target organs in oral repeated dose toxicity studies included in the safety evaluation reports

As the 90-day oral RDT studies are specifically designed to provide information on toxic effects and indicate target organs upon repeated exposure (OECD 2018), information on target organs and critical effects have primarily been retrieved from those studies. Exceptions include the nine opinions previously mentioned where the data described in the opinions were provided from open literature and through read-across from other oral studies. Of the 88 opinions containing oral RDT data (listed in Online Resource 1), eight cosmetic ingredients were devoid of adverse effects at the maximum allowed test dose (1000 mg/kg bodyweight) resulting in the determination of an NOAEL and a no observed effect level (NOEL) at this dose for 6 and 2 ingredients, respectively, while an NOAEL was set at the highest tested dose (< 1000 mg/kg bodyweight) for five cosmetic ingredients. Consequently, based on the lack of observed adverse effects, it was not possible to identify the target organ for these 13 compounds.

Critical effects and/or target organs used to set an NOAEL were identified through RDT studies in 75 opinions. Among these 75 opinions, the liver was identified as the target organ for 20 cosmetic ingredients, based on alterations in blood biochemistry, including enzyme activities, and on histopathological changes (e.g., vacuolisation and necrosis), making it one of the most frequent targets of toxicity. Due to its anatomical proximity to the digestive tract and high blood flow rate, together with its key role in the biotransformation of xenobiotics, it is not surprising that the liver appears as a main target of toxicity upon repeated oral exposure (Hayes and Kruger 2014).

Based on aberrant haematological parameters such as number of blood cells, amount of haemoglobin, and morphological and/or histopathological changes in the spleen, the haematological system and/or the spleen, often in combination and, therefore, paired as one organ system, were found to be commonly affected. The haematological system is complex, multicellular and in a dynamic state of cell proliferation, differentiation, activation, and maturation. The spleen is responsible for red blood cell production and removal of damaged blood cells (Hayes and Kruger 2014; Mebius and Kraal 2005). Hence, it is conceivable to assume that the turnover and alteration of cells in the blood, as well as the high blood flow through the spleen, makes the system vulnerable to chemical-induced systemic toxicity.

**Fig. 1:**
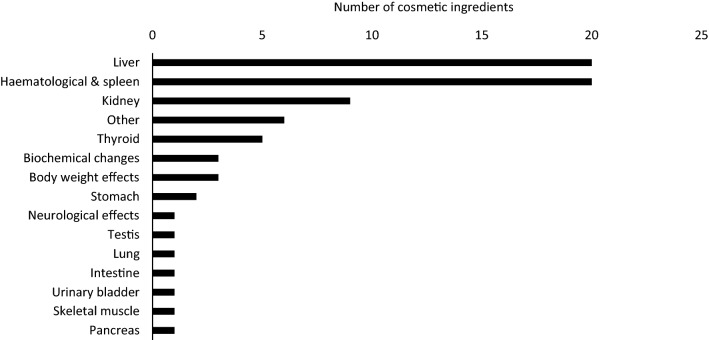
Identification of target organs and critical effects of 75 cosmetic ingredients from oral repeated dose toxicity studies described in the safety evaluation reports issued between 2009 and 2019

The third most affected organs, although with markedly lower occurrence compared to the liver and the haematological system, were demonstrated to be the kidneys. In most cases, this was based on histopathological changes (Fig. 1) such as cellular degeneration (3) and the presence of hyaline droplets (3) in the renal tubules. However, for the majority of cases, the study descriptions in the SCCS opinions did not allow to determine which part of the tubules were affected. Additionally, hyaline droplets are often seen as a non-specific response and thus of limited relevance to humans, especially when exclusively observed in male rats, which was the case for two out of  the three compounds. The kidneys are responsible for maintaining a consistent internal environment by regulating the body’s salt, water, and acid–base balance. This is achieved through blood filtration, approximately 25% of the cardiac output, and excretion of waste products (Barnett and Cummings 2019). Moreover, the epithelial cells lining the proximal tubules contain a plethora of transport proteins, often leading to accumulation of chemical compounds, resulting in a high concentration of chemicals within the tubular epithelial cells relative to that in plasma (Barnett and Cummings 2019; Hayes and Kruger 2014) which may partly explain the cellular degeneration observed in the renal tubules. Collectively, these properties make the kidneys sensitive to toxicants and, thus, not an unexpected target for systemic toxicity.

### Identification of morphological, histopathological, and biochemical changes related to hepatotoxicity in oral repeated dose toxicity studies described in the safety evaluation reports

**Fig. 2:**
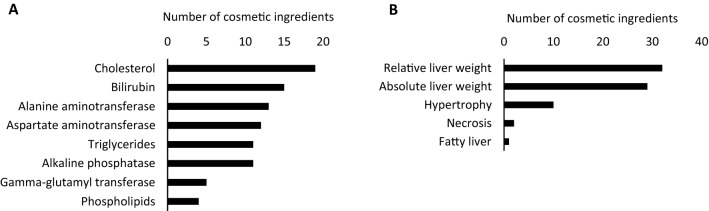
**a** Identification of changes in clinical parameters possibly linked to hepatotoxicity in oral repeated dose toxicity studies described for the 49 liver-affecting cosmetic ingredients in safety evaluation reports issued between 2009 and 2019. **b** Identification of morphological and histopathological changes possibly linked to hepatotoxicity in oral repeated dose toxicity studies described for the 49 liver-affecting cosmetic ingredients in safety evaluation reports issued between 2009 and 2019

Following the identification of the liver as the main target for toxicity in oral RDT studies, the relevance of this finding was further investigated by listing potentially toxicological parameters related to hepatotoxicity as described in the SCCS opinions (Fig. 2a and b). In addition to the 20 cosmetic ingredients demonstrated to elicit a critical effect in the liver, 29 additional ingredients of the 88 opinions containing oral RDT data also altered one or more parameters related to hepatotoxicity, resulting in 49 liver-affecting compounds in total. For 29 of the 88 cosmetic ingredients, a combination of changes in parameters related to hepatotoxicity and haematotoxicity was seen. Alterations in serum cholesterol and bilirubin levels were the most commonly observed biochemical modifications (Fig. 2a), while altered liver weight represented the most frequently occurring morphological change (Fig. 2b). Deviating levels of one or more liver enzymes were also commonly seen (Fig. 2a), i.e., for 28 substances in total. However, it should be noted that these safety evaluations have not been designed for this specific purpose and the relevance of observed alterations in individual parameters for characterising liver injury must be seen as limited. For example, hepatocellular hypertrophy can reflect adaptive responses to a compound due to, e.g., enzyme induction (Hall et al. 2012). Changes in liver weight can be attributed either to transient adaptive effects or an unfavourable taste of the compound causing a reduced food intake with subsequent reduction in body weight of the test animals, thereby also affecting organ weight (Cattley and Cullen 2013; Vinken et al. 2012). Unaccompanied, many of these deviations are not necessarily regarded as toxicologically relevant and do not always imply adversity. Yet, elevated enzyme activities such as alanine aminotransferase (ALT) and aspartate aminotransferase (AST) reflect hepatocyte damage and may provide a first indication of liver toxicity (David and Hamilton 2010). Consequently, changes in individual parameters have rather limited significance for characterising potential (liver) injury. To accurately identify adversities, a battery of tests that detect clinically important biochemical, histopathological, and morphological parameters needs to be considered.

Certain combinations of effects might, however, be indicative of specific adverse effects. Concomitant increases in serum AST, cholesterol, and triglycerides, alongside fat accumulation in hepatocytes, resulting from impaired balance between the rate of synthesis and the release of triglycerides from the hepatocytes, could imply steatosis-inducing potential of a compound (Hayes and Kruger 2014; Ipsen et al. 2018). Likewise, simultaneous increases in serum alkaline phosphatase (ALP), gamma-glutamyl transferase (GGT), and bilirubin, in addition to hepatocellular necrosis, are predominant features of cholestatic liver injury, i.e., hepatocellular accumulation of bile (Boone et al. 2005; Greim 2008; Hayes and Kruger 2014; Robles-Diaz et al. 2015). In this context, several compounds covering a wide chemical space and various application areas have been found to induce liver steatotic and cholestatic effects (Al-Eryani et al 2015; Vilas-Boas et al. 2019). Cosmetic ingredients provoking a change in one or more parameters potentially associated with steatosis and cholestasis are depicted in Tables 2 and 3, respectively. Based on these combined observations, none of the 49 cosmetic ingredients triggered changes in the combination of parameters related to steatosis to a degree prospectively raising concern, i.e., affecting three out of four parameters (Vinken et al. 2012). Two out of the 49 liver-affecting cosmetic ingredients were found to alter three or more of the parameters associated with cholestasis, namely 2,7-naphthalenediol and Basic red 51, and could, therefore, be considered as plausible cholestasis-inducing compounds (SCCS 2010a, 2011).

**Table 2:** Identification of cosmetic ingredients provoking a change in at least one parameter associated with steatosis based upon alterations observed in oral repeated dose toxicity studies described for the 49 liver-affecting cosmetic ingredients in safety evaluation reports issued between 2009 and 2019

Cosmetic ingredient	Function	↑AST*	↑TG**	↑Cholesterol	Hepatic fat accumulation
5-Amino-6-chloro-o-cresol	Hair dyeing ingredient used as a precursor for hair dyeing products. The reaction can be accelerated by the addition of an oxidising agent (e.g., hydrogen peroxide), but can also be achieved by air oxidation			x	
Acetylated vetiver oil—AVO	Used as a fragrance in perfumes and cosmetics			x	
Basic brown 17	Hair dyeing ingredient used as a direct dye for hair colouring products		x	x	
Basic red 51	Hair dyeing ingredient used in direct hair dye formulations and in oxidative hair dyes after mixing with the oxidative agent		x	x	
Basic violet 2	Hair dyeing ingredient used as a non-reactive hair colouring agent in non-oxidative hair dye-formulation and oxidative hair dye formulations. Also used as a colourant (CI 42,520) in cosmetic products intended to come into contact only briefly with the skin			x	
Bis(butylbenzoate) diaminotriazine aminopropyltrisiloxane	Used as a UV filter (*λ*_max_ = 310 nm) proposed to be used in sunscreen formulations at a maximum concentration of 10%	x			
Butylphenyl methylpropional (*p*-BMHCA)	Used as a fragrance ingredient in many compounds for cosmetic products as well as in non-cosmetic products such as household cleaners and detergents				x
Cetylpyridinium chloride	Used as a disinfectant in mouthwashes cosmetic products up to a concentration of 0.1%, all other oral hygiene cosmetic products up to a concentration of 0.5%, skin lotions and creams up to a concentration of 0.2% and anti-perspirant deodorants up to a concentration of 2.0%	x			
Decamethylcyclopentasiloxane D5	Used as volatile excipient in cosmetic products. It can have many different functions in cosmetic products including antistatic, emollient, humectant, solvent, viscosity controlling, and hair conditioning				x
EcoG +	Used as a preservative in the internal parts of packaging containers (i.e., the parts of the packaging in direct contact with the cosmetic product)			x	
HC blue 15	Hair dyeing ingredient used in oxidative hair dyes as a non-reacting component		x	x	
HC yellow 13	Hair dyeing ingredient used as a hair colouring agent ("direct dye") in non-oxidative and oxidative hair dye formulations			x	
Hydroxyethyl-2-nitro-*p*-toluidine	Hair dyeing ingredient used as a direct dye in hair dye formulations and as a non-reactive dye in oxidative hair dye formulations	x			
Hydroxyethyl-3,4-methylenedioxyaniline HCl	Hair dyeing ingredient used in oxidative hair dye formulations after mixing with the developer containing hydrogen peroxide			x	
Hydroxyethyl-*p*-phenylenediamine sulphate	Hair dyeing ingredient used in oxidative hair dye formulations			x	
Hydroxypropyl *p*-phenylenediamine and its dihydrochloride salt (A165)	Hair dyeing ingredient used in oxidative hair dye formulations	x		x	
Methylimidazoliumpropyl *p*-phenylenediamine HCl (A166)	Hair dyeing ingredient used in oxidative hair dye formulations	x			
*N*,*N*'-bis-(2-hydroxyethyl)-2-nitro-*p*-phenylenediamine	Hair dyeing ingredient used as a direct hair dye for hair colouring products. Used in oxidative hair dye formulations with and without mixing with an oxidising agent (e.g., hydrogen peroxide)		x	x	
*N*-Methyl-2-pyrrolidone	Used as a solvent and a surfactant in cosmetic products			x	
*o*-Aminophenol	Hair dyeing ingredient used in oxidative hair dye formulations	x			
Phenoxyethanol	Used as a preservative in cosmetic formulations at a maximum concentration of 1.0%		x		
Toluene-2,5-diamine (sulphate)	Hair dyeing ingredient used in oxidative hair dye formulations (precursor)	x			
Vetiveryl acetate	Used as a fragrance in perfumes and cosmetics			x	

*Aspartate aminotransferase

**Triglycerides

**Table 3:** Identification of cosmetic ingredients provoking a change in at least one parameter associated with cholestasis based on alterations observed in oral repeated dose toxicity studies described for the 49 liver-affecting cosmetic ingredients in safety evaluation reports issued between 2009 and 2019

Cosmetic ingredient	Function	↑ALP*	↑GGT*	↑Bilirubin	Hepatocellular necrosis
1,5-Naphthalenediol	Hair dyeing ingredient used in oxidative and non-oxidative hair dye formulations			x	
2,6-Dihydroxyethylaminotoluene	Hair dyeing ingredient used as a precursor for hair colours. It reacts with primary intermediates to form the final dye-stuff. The reaction can be accelerated by the addition of an oxidising agent (e.g., hydrogen peroxide), but it can also be achieved by air oxidation			x	
2,7-Naphthalenediol	Used in oxidative and non-oxidative hair dye formulations with a maximum on-head concentration of 1%		x	x	x
5-Amino-6-chloro-*o*-cresol	Used as a precursor for hair dyeing products. The reaction can be accelerated by the addition of an oxidising agent (e.g., hydrogen peroxide), but can also be achieved by air oxidation			x	
Basic brown 17	Hair dyeing ingredient used as a direct dye for hair colouring products		x	x	
Basic red 51	Hair dyeing ingredient used in direct hair dye formulations and oxidative hair dyes after mixing with the oxidative agent	x	x		x
Citric acid (and) silver citrate	Used as a preservative system in aqueous leave-on and rinse-off cosmetic products. Citric acid and silver citrate is used in deodorants	x			
Diethylene glycol monoethyl ether	Used in cosmetics and dermatological preparations and as a solvent in some medicine products. Its physical properties make DEGEE useful to solubilise lipophilic and hydrophilic compounds. Moreover, DEGEE enhances the percutaneous absorption through the skin and mucosal barriers	x			
EcoG +	Used as a preservative in the internal parts of packaging containers (i.e., the parts of the packaging in direct contact with the cosmetic product)	x			
Hydroxyethyl-2-nitro-*p*-toluidine	Used as a direct dye in hair dye formulations at a maximum concentration of 1% and as a non-reactive dye in oxidative hair dye formulations at a maximum concentration of 1%, after dilution with the oxidative agent	x		x	
Hydroxyethyl-3,4-methylenedioxyaniline HCl	Hair dyeing ingredient used in oxidative hair dye formulations after mixing with the developer containing hydrogen peroxide			x	
Hydroxypropyl *p*-phenylenediamine and its dihydrochloride salt (A165)	Hair dyeing ingredient used in oxidative hair dye formulations		x	x	
Methoxypropylamino cyclohexenylidene ethoxyethylcyanoacetate (S87)	Used as a UV filter in personal care products, including sun care cosmetic formulations at a maximum concentration of 3% w/w			x	
*N*-Methyl-2-pyrrolidone	Used as a solvent and a surfactant in cosmetic products	x			
Phenoxyethanol	Used as a preservative in cosmetic formulations at a maximum concentration of 1.0%	x			

*Alkaline phosphatase

**Gamma-glutamyl transferase

### Identification of morphological, histopathological, and biochemical changes related to the haematological system and the spleen in oral repeated dose toxicity studies described in the safety evaluations

**Fig. 3:**
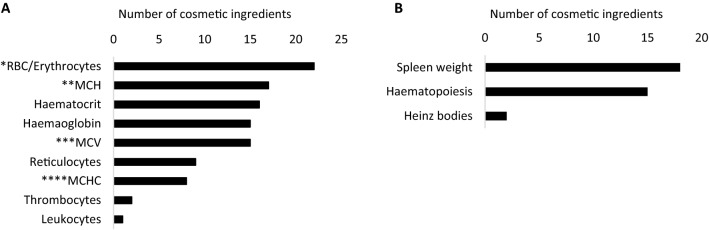
**a** Identification of haematological changes possibly linked to toxic effects in the haematological system as described in oral repeated dose toxicity studies for the 42 haematology-affecting cosmetic ingredients in safety evaluation reports issued between 2009 and 2019. **b** Identification of morphological and histopathological changes possibly linked to toxic effects in the spleen described in oral repeated dose toxicity studies for the 42 haematology-affecting cosmetic ingredients in safety evaluation reports issued between 2009 and 2019. *Red blood cells. **Mean corpuscular haemoglobin. ***Mean corpuscular volume. ****Mean corpuscular haemoglobin concentration

As holds for the liver, the parameters possibly related to haematotoxicity described in the SCCS opinions were listed. In total, 42 of the 88 cosmetic ingredients induced changes in one or more parameters associated with the haematological system following oral RDT testing. The most frequently observed morphological and histopathological changes in the spleen were alterations in weight and occurrence of haematopoiesis (Fig. 3b). While both effects can be caused by a large variety of insults, the former is considered as a sensitive indicator of immune toxicity (Kim 2010; Michael et al. 2007). The most commonly affected haematological parameters were changes in red blood cell count (RBC), mean concentration of haemoglobin (MCH) in red blood cells, and haematocrit (i.e., the ratio of the volume of red blood cells to the total blood volume) (Fig. 3a). When decreased, all these parameters are indicative of anaemia, albeit a correct diagnosis requires a more complex assessment (Broadway-Duren and Klaassen 2013; Cascio and DeLoughery 2017) with additional tests generally not included in RDT studies. Commonly analysed parameters related to the haematological system can be used to classify the suspected anaemia as regenerative or non-regenerative (Grimes and Fry 2015). The former encompasses reduced levels of circulating red blood cells and/or other red blood cell parameters such as haemoglobin and haematocrit, which is accompanied by an increase in immature blood cells, i.e., reticulocytes. If the regenerative response is potent enough to counteract the decreased levels of red blood cells, a regenerative anaemia can be diagnosed based on increased erythrocyte mean cell volume (MCV) and subnormal mean corpuscular haemoglobin concentration (MCHC). On the contrary, in non-regenerative anaemia, no increase in the levels of reticulocytes is seen. Non-regenerative anaemia occurs less often as a result of primary haematotoxicity, but is more frequently a complication of various non-haematologic diseases, e.g., inflammation (Grimes and Fry 2015). In Table 4, the cosmetic ingredients that provoked changes in the above-mentioned haematological parameters are listed. Alterations indicative of non-regenerative anaemia (i.e., anaemia with no concomitant increase in reticulocytes) were observed for 2,7-naphthalenediol, erythrosine (CI 45430), disperse violet 1 (1,4-diamino-anthraquinone), HC red 7, and sodium perborate and perboric acid. This is in line with the finding that 2,7-naphthalenediol, erythrosine (CI 45430), and disperse violet 1 (1,4-diamino-anthraquinone) are believed to target organs other than the haematological system (Online Resource 1). Conversely, HC red 7, and sodium perborate and perboric acid are believed to target the haematological system and may induce anaemia only as a secondary effect. Again, it is important to keep in mind that these safety evaluation reports of cosmetic ingredients have not been designed to diagnose specific diseases, but rather to describe general toxicity, and thus, the results need to be considered with caution.

**Table 4:** Identification of cosmetic ingredients provoking a change in parameters commonly used for the diagnosis of anaemia based on alterations observed in oral repeated dose toxicity studies described for the 42 haematology-affecting cosmetic ingredients in safety evaluation reports issued between 2009 and 2019

Cosmetic ingredient	Function	↓Haematocrit/RBC/mean haemoglobin*	↑MCV**	↓MCHC***	↑Reticulocytes
1-Hexyl 4,5-diamino pyrazole sulphate	Hair dyeing ingredient used as an oxidative hair colouring agent (precursor). The oxidative colouring agent and the developer are mixed at a ratio of 1 + 1 to 1 + 3	x	x		x
1-Hydroxyethyl-4,5-diamino pyrazole sulphate	Hair dyeing ingredient used in oxidative hair dye formulations after mixing with peroxide		x		
2,6-Diaminopyridine	Hair dyeing ingredient used in oxidative hair colouring products after mixing in a 1:1 ratio with hydrogen peroxide just prior to use	x	x		x
2,7-Naphthalenediol	Hair dyeing ingredient used in oxidative and non-oxidative hair dye formulations	x			
2-Amino-4-hydroxyethylaminoanisole sulphate	Hair dyeing ingredient used in oxidative hair dye formulations after mixing the developer containing oxidative agent	x			x
4-Chlorooresorcinol	Hair dyeing ingredient used as a coupler in oxidative hair dye formulations	x			x
4-Nitrophenyl aminoethylurea	Hair dyeing ingredient used as direct dye in semi-permanent hair formulations and as a hair colouring agent (direct dye) in oxidative hair dye formulations	x			x
5-Amino-6-chloro-*o*-cresol	Hair dyeing ingredient used as a precursor for hair dyeing products	x	x	x	
Acid black 1	Hair dyeing ingredient used as a direct hair colouring agent in non-oxidative hair dye formulations	x			x
Acid orange 7	Hair dyeing ingredient used as a direct hair colouring agent in non-oxidative as well as in oxidative hair dye formulations				x
Basic red 76	Hair dyeing ingredient used as a direct dye for hair colouring products	x			x
Basic violet 2	Hair dyeing ingredient used as a non-reactive hair colouring agent in non-oxidative hair dye-formulation and in oxidative hair dye formulations. Also used as a colourant in cosmetic products intended to come into contact only briefly with the skin	x	x		
Basic yellow 57	Hair dyeing ingredient used as a direct dye for hair colouring products	x			x
CI 45,430, erythrosine	Used as a red colour additive in cosmetics	x			
Disperse violet 1 (1,4-diamino-anthraquinone)	Hair dyeing ingredient used as a hair colour in semi-permanent hair dye formulations	x			
HC red 13	Hair dyeing ingredient used as a non-reactive hair colouring agent (“direct dye”) in semi-permanent hair dye formulations and oxidation hair dye formulations	x		x	
HC red 7	Hair dyeing ingredient used in semi-permanent hair dye formulations	x			
HC yellow 2	Hair dyeing ingredient used in non-oxidative hair dye-formulation and oxidative formulations	x	x	x	
HC yellow 7	Hair dyeing ingredient used in semi-permanent hair dye formulations	x		x	
Hydroxyethyl-3,4-methylenedioxyaniline HCl	Hair dyeing ingredient used in oxidative hair dye formulations after mixing with the developer containing hydrogen peroxide	x			x
Methoxypropylamino cyclohexenylidene ethoxyethylcyanoacetate (S87)	Used as a UV filter in personal care products, including sun care cosmetic formulations at a maximum concentration of 3% w/w	x		x	x
Picramic acid and sodium picramate	Hair dyeing ingredient used as a direct hair colouring agent in non-oxidative as well as in oxidative hair dye formulations	x	x	x	x
Sodium perborate and perboric acid	Hair dyeing ingredient used in oxidative hair colouring products after mixing with water just prior to use	x			

*Red blood cell count

**Mean corpuscular volume

***Mean corpuscular haemoglobin concentration

## Conclusion

The goal of the current study was to identify the main target organs and/or critical effects as well as to characterise the manifestations of toxicity of cosmetic ingredients in these organs in oral RDT studies. RDT data were collected from safety evaluation reports published by the SCCS between 2009 and 2019. Not surprisingly, it was found that the most frequently affected organs by cosmetic ingredients were the liver and the haematological system, whose inherent physiological functions make them susceptible to toxicants (Cattley and Cullen 2013; Hayes and Kruger 2014; Mebius and Kraal 2005). It is well known that drug-induced liver injury remains a common cause of the withdrawal of drugs in pre-clinical and even clinical phases of drug development (Lee 2013; Onakpoya et al., 2019). It has also been reported that chemicals from various other application areas could trigger liver adversities such as steatosis and cholestasis (Al-Eryani et al. 2015; Vilas-Boas et al. 2019). Here, for cosmetic ingredients, certain combinations of changes in relevant parameters that could indicate liver steatosis and cholestasis were collected (Tables 2 and 3) for all potentially liver-affecting compounds (49). The liver’s complexity and diverse functions, in combination with its varied response to injury, require careful consideration of multiple parameters to ensure scientifically valid results. It should be further stressed that the parameters available in the SCCS opinions constitute only a limited part of the clinically relevant information addressed to diagnose liver steatosis and cholestasis. This also holds true for possible adverse effects associated with the haematological system. The identification of the kidneys as the third most commonly affected organs was based on various histopathological changes for which no clear pattern could be identified. Therefore, characterisation of the toxic effects is limited to the observation that the most prominent alterations were the presence of hyaline droplets and cellular degeneration in the renal tubules. As previously mentioned, the studies used in the safety evaluation reports are not designed to investigate specific adverse effects, but rather indicate a general capability of a compound to induce harmful effects. Therefore, it is of utmost importance to interpret the findings presented here with caution to avoid incorrect conclusions. Nevertheless, two cosmetic ingredients, i.e., 2,7-naphthalenediol and Basic red 51, were demonstrated to affect three or more parameters associated with cholestasis, therefore possibly indicating a cholestasis-inducing potential (SCCS 2010a, 2011). Moreover, HC red 7, and sodium perborate and perboric acid provoked changes indicative of indirect haematotoxic effects. Indeed, in the safety evaluation of HC red 7, additional effects are mentioned that could be of interest for further investigations (SCCS 2009). However, the studies in the safety evaluation of sodium perborate and perboric acid are old and poorly described; therefore, no conclusions can be drawn in this regard (SCCS 2010b). Overall, the outcome of the present study is in line with a screening of SCCS opinions published between 2000 and 2009 (Vinken et al. 2012). In fact, both 2,7-naphthalenediol and Basic red 51 were previously identified as plausible cholestasis-inducing compounds, meaning that they have been evaluated multiple times (Vinken et al. 2012). In this respect, repeated evaluations of a compound are a common event, as 74 of the total number of compounds (101) included in the current screening have been addressed by the SCCS, or its predecessors, more than once. There are various reasons for the re-evaluation of a cosmetic ingredient, for instance due to inadequate provision of data for safety evaluation in the first submission or due to a request from the industry for an extension of the allowed use concentration of the specific ingredient.

Since 2013, the animal testing ban has largely impeded the marketing of new cosmetic ingredients. This innovation issue will remain until there are suitable non-animal methods that address the most complex human toxicological endpoints that historically have been evaluated with animal RDT testing. Due to the complex interactions in a whole organism, it is very unlikely to find a one-to-one conversion from one in vivo to one in vitro test as replacement of RDT testing. Instead, it is envisaged that human-based in silico*, *in vitro, and in chemico approaches, collectively known as new approach methodologies (NAMs), will comprise the next generation risk assessment (NGRA). Although scientific challenges remain to be tackled before NAMs are used by default in safety evaluations and advantages with their use, such as an increased relevance to human health, becomes evident. Indeed, NAMs have shown great promise to provide a large amount of data to fill information gaps in both hazard and exposure assessments (Rogiers et al. 2020). NGRA is anticipated to be hypothesis-driven and exposure-led, including multiple NAMs which are integrated and combined in novel approaches (Dent et al. 2018). Methods such as exposure-based waiving using thresholds of toxicological concern (TTC) for structurally related compounds and chemicals belonging to a defined chemical space may be satisfactory in case of very limited exposures (Kroes et al. 2007). Grouping of chemicals for read-across purposes can be performed at different levels of biological activity (e.g., common target organ, common critical effect, or mode of action) (Bal-Price and Meek 2017). Information about the effects of cosmetic ingredients on target organs will facilitate the development of NAMs, as they should be challenged with cosmetic ingredients as a proof-of-concept exercise, thereby making information on expected target organs and critical effect(s) essential. Hence, the results of this study call for prioritising the development of NAMs suitable to address biological activity and toxicity provoked by cosmetic ingredients in the liver and the haematological system.

## Electronic supplementary material

Below is the link to the electronic supplementary material.

Supplementary file1 (XLSX 27 kb)

## Data Availability

The datasets generated during and/or analysed during the current study are available from the corresponding author on reasonable request.

## Abbreviations

ALT: Alanine aminotransferase
ALP: Alkaline phosphatase
AST: Aspartate aminotransferase
EU ToxRisk: Integrated European ‘Flagship’ Programme Driving Mechanism-based Toxicity Testing and Risk Assessment for the twenty-first century
GGT: Gamma-glutamyl transferase
MCH: Mean corpuscular haemoglobin
MCHC: Mean corpuscular haemoglobin concentration
MCV: Mean cell volume
MoS: Margin of safety
NAM(s): New approach methodology(ies)
NGRA: Next-generation risk assessment
NOAEL: No observed adverse effect level
NOEL: No observed effect level
POD(_sys_): Point of departure (_systemic_)
RBC: Red blood cell count
RDT: Repeated dose toxicity
SCCS: Scientific Committee on Consumer Safety
SED: Systemic exposure dose
SEURAT: Safety Evaluation Ultimately Replacing Animal Testing
TTC: Threshold of toxicological concern

## Footnotes

1 OECD guidelines are available via https://www.oecd.org/env/ehs/testing/oecdguidelinesforthetestingofchemicals.htm.

2 More information regarding the project is available via https://www.seurat-1.eu/.

3 More information regarding the project is available via https://www.eu-toxrisk.eu/.

4 SCCS opinions are available for download via https://ec.europa.eu/health/scientific_committees/consumer_safety_en.

## Code availability

Microsoft^®^ Office Excel version 16.10 (180210).
